# Effect of transporting an evidence-based, violence prevention intervention to Jamaican preschools on teacher and class-wide child behaviour: a cluster randomised trial

**DOI:** 10.1017/gmh.2017.29

**Published:** 2018-02-19

**Authors:** H. Baker-Henningham, S. Walker

**Affiliations:** 1 School of Psychology, Bangor University, Bangor, UK; 2 Caribbean Institute for Health Research, University of the West Indies, Kingston §7, Jamaica

**Keywords:** Child behaviour, cultural adaptation, evidence-based intervention, interventions, preschool, violence prevention

## Abstract

**Introduction.:**

Based on extensive piloting work, we adapted the Incredible Years (IY) teacher-training programme to the Jamaican preschool setting and evaluated this adapted version through a cluster-randomised trial.

**Methods.:**

Twenty-four community preschools in Kingston, Jamaica were randomly assigned to intervention (12 schools, 37 teachers) or control (12 schools, 36 teachers). The intervention involved training teachers in classroom management through eight full-day training workshops and four individual 1-h in-class support sessions. Outcome measurements included direct observation of teachers’ positive and negative behaviours to the whole class and to high-risk children and four observer ratings: two measures of class-wide child behaviour and two measures of classroom atmosphere. Measures were repeated at a six-month follow-up.

**Results.:**

Significant benefits of intervention were found for teachers’ positive [effect size (ES) = 3.35] and negative (ES = 1.29) behaviours to the whole class and to high-risk children (positive: ES = 0.83; negative: ES = 0.50) and for observer ratings of class-wide child behaviour (ES = 0.73), child interest and enthusiasm (ES = 0.98), teacher warmth (ES = 2.03) and opportunities provided to share and help (ES = 5.72). At 6-month follow-up, significant benefits of intervention were sustained: positive behaviours (ES = 2.70), negative behaviours (ES = 0.98), child behaviour (ES = 0.50), child interest and enthusiasm (ES = 0.78), teacher warmth (ES = 0.91), opportunities to share and help (ES = 1.42).

**Conclusions.:**

The adapted IY teacher-training programme produced large benefits to teacher's behaviour and to class-wide measures of children's behaviour, which were sustained at 6-month follow-up. Benefits were of a similar magnitude to those found in a pilot study of the minimally adapted version that required significantly more in-class support for teachers.

## Introduction

School and societal violence is an enormous problem in Jamaica and the wider Caribbean and the primary prevention of violence is a national and regional public health priority (Meeks-Gardner *et al.* 2005). Violence is also recognised as a leading global public health problem and makes a substantial contribution to morbidity and mortality with high social and economic costs (WHO, 2014). Juvenile delinquency and antisocial behaviour in adulthood can often be traced back to early childhood. Young children who have conduct problems (which describes a range of antisocial behaviours including oppositional, aggressive, dishonest and disruptive behaviours) are not only more likely to have continued antisocial and aggressive behaviour in later childhood and adolescence, but they are more likely to experience academic failure and early school drop-out and to engage in crime and violence in adulthood (Moffitt & Scott, 2008). Interventions to prevent and reduce conduct problems in early childhood are thus an integral component of violence prevention programmes.

Schools offer a logical setting for implementing preventative interventions for young children, and school-based violence prevention programmes show significant benefits to children's violent and aggressive behaviour in high-income countries (Hahn *et al.* 2007; Wilson & Lipsey, 2007). One type of violence prevention programme involves training teachers in classroom behaviour management, (for example, how to create a nurturing and supportive classroom environment, how to prevent and manage child behaviour problems and how to promote children's social and emotional competence), and a recent meta-analysis showed that these programs, when implemented in primary school, benefit multiple child outcomes including academic achievement, behaviour, social–emotional skills and motivation (Korpershoek *et al.* 2016). Benefits of such interventions have also been found in pre-primary contexts: a meta-analysis of interventions focused on training caregivers in centre-based services for preschool children found benefits to caregiver–child interaction, classroom quality and child social–emotional development (Werner *et al.* 2016). Training staff in early childhood centres in low and middle-income countries (LMIC) may be particularly important given the evidence that aggression and disruptive behaviour can increase on exposure to poorer quality preschool environments (Aboud, 2006; Bernal & Fernandez, 2013). There is limited evidence of such interventions from LMIC (Baker-Henningham, 2014) where schools often have less well-trained teachers and lower levels of resources.

We conducted a pilot study of the Incredible Years (IY) teacher-training programme and a curriculum unit on social–emotional skills in Jamaican preschools and found benefits to teacher practices and classroom atmosphere (Baker-Henningham *et al.* 2009a) and to teacher reported behaviour of children with initial higher levels of behaviour problems (Baker-Henningham *et al.* 2009b). In-depth interviews with teachers showed that the intervention was acceptable to teachers (Baker-Henningham & Walker, 2009). Throughout the pilot study, we documented teachers’ engagement with the programme on an ongoing basis. During each workshop, the co-facilitator recorded information including: (i) how teachers responded to the new material, (ii) any difficulties teachers showed in understanding and performing the skills taught and (iii) teachers’ feedback on their use of strategies. The facilitator and co-facilitator also completed self and peer-evaluations of each workshop that included detailed reflections of teachers’ engagement with the material. In addition, after each in-class support session, the coach documented the strengths and needs of each individual teacher based on her own observations and her discussions with the teacher. These data, combined with the data from the qualitative and quantitative evaluation, were used to adapt the IY teacher-training programme to enhance its relevance to teachers working in low-resource settings. The content was similar to the original programme but significant adaptations were made to the materials and the method of delivery of the intervention. Further details of the rationale for adapting the programme, the source of the evidence underpinning this rationale and the nature of the adaptations made are given in Table 1. This adapted programme was evaluated, in a cluster-randomised trial in 24 preschools, for its effect on the behaviour of children who were at high risk for developing conduct problems. Significant benefits were found in terms of reduced conduct problems by independent observations [effect size (ES) = 0.42], teacher report (ES = 0.47) and parent report (ES = 0.22); increased social skills by independent observations (ES = 0.74) and teacher report (ES = 0.59) and increased school attendance (ES = 0.30) (Baker-Henningham *et al.* 2012).

**Table 1.** Adaptations made to the Incredible Years teacher-training programme based on the pilot study

Rationale for adaptation	Source of the evidenceSource of the evidence	Adaptations to materials
Video vignettes were from US schools and the classroom context was unfamiliar to the teachers (e.g. higher teacher–child ratio, more resources, better physical conditions). Significant amounts of time were spent discussing, role-playing and modelling how the strategies could be implemented in the Jamaican context	Facilitator reflections on workshop. Co-facilitator notes on workshop. Coach's notes on the in-class support sessions	Make video vignettes of teachers in Jamaica preschool classrooms using appropriate behaviour management techniques
Teachers lost concentration and focus during longer video vignettes, teachers often praised vignettes with negative practices and some video examples were very subtle and easily missed by the teachers	Facilitator reflections on workshop. Co-facilitator notes on workshop	Make only short vignettes (1–2 min). Use positive role models only Make the video vignettes very explicit
Teachers did not always have sufficient prior knowledge to easily grasp the strategies as presented in the original programme; some content needed to be repeated several times	Facilitator reflections on workshop. Co-facilitator notes on workshop	Design activities to help teachers acquire new knowledge (e.g. card-sorting activity to differentiate between good and bad instructions). Provide handouts with specific examples of how to use a particular strategy. Design role plays to introduce and reinforce new concepts
Teachers varied in their motivation, competence and persistence in utilising the strategies	Coach's notes on the in-class support sessions. Teacher feedback during workshop. Qualitative evaluation of pilot study	(i) Provide easy to use checklists for teachers to monitor their progress, (ii) Provide simple handouts clarifying key points and (iii) Design classroom assignments to encourage teachers to document their use of specific skills and their effects on individual children (so teachers recognise the effectiveness of the strategies)
Implementing the strategies was difficult in classrooms characterised by overcrowding, few resources, high noise levels and high child/staff ratios	Teacher feedback during workshop. Coach's notes on in-class support sessions. Qualitative evaluation of pilot study	Design new brainstorms and small group activities to help teachers generate and practice solutions to common problems. Provide handouts with creative ideas for solving common problems
Some teachers needed substantial assistance before they were competent at using the strategies without help	Facilitator reflections on workshop. Coach's notes on the in-class support sessions	Design step-by-step guidance on how to explicitly teach skills to the children (e.g. following classroom rules, using social skills) with accompanying checklists for teachers to follow
Teachers found it difficult to find time to utilise a curriculum unit on social and emotional competence containing structured lessons	Quantitative and qualitative evaluation of the pilot study	Design classroom activities that integrate the teaching of social and emotional skills into everyday teaching and learning activities
Rationale for adaptation	Data used	Adaptations to methods
Teachers preferred to spend less time in passive activities (e.g. watching video vignettes, group discussions) and more time in active activities (e.g. role plays, group work)	Facilitator reflections on workshop. Co-facilitator notes on workshop. Qualitative evaluation of pilot study	Reduce the number of video vignettes and the time spent on group discussion. Spend more time on active, hands-on activities (e.g. role plays and small group work)
Teachers often lacked confidence to try the strategies in their classroom without assistance and/or became disheartened if the strategies did not work as intended	Teacher feedback during workshop. Co-facilitator notes on workshop. Qualitative evaluation of pilot study	Spend more time on role plays and group activities to give teachers practice using the strategies during the workshops. Provide structured in-class support sessions that focus on modelling and prompting teachers, providing supportive feedback and assisting with problem solving
Teachers found it difficult to generalise their use of the strategies to different situations in the classroom	Teacher feedback during workshop. Coach's notes on the in-class support sessions	Role play using each strategy in different situations. Provide opportunity for teachers to work in small groups to plan and practice how they will use a strategy to address different child behaviours and across different activities in the school day
Teachers were unaccustomed to spending time playing with children, engaging in social conversation and following children's lead. There was a strong emphasis on academic learning with little or no focus on promoting children's social and emotional skills	Quantitative and qualitative evaluation of pilot study	Increase the time allocated to the topics ‘Building positive relationships with children’ and ‘Praise and rewards’. Increase the length of the workshops (from 5–6 days to 8 days)
Some teachers needed more guidance before they could use the strategies competently and consistently	Facilitator reflections on workshop. Coach's notes on the in-class support sessions	Utilise demonstrations and explicit teaching in addition to using the more collaborative approach advocated by the IY programme

In this paper, we report the effects of this intervention on (i) teachers’ interactions with the whole class, (ii) teachers’ interactions with children at high risk for developing conduct problems, (iii) the classroom atmosphere and (iv) the class-wide child behaviour. We also examined whether benefits are sustained at a 6-month follow-up. Finally, we compare this intervention, which has been extensively adapted to the Jamaican preschool context, to the intervention used in the previous study where only minor adaptations were made.

## Methods

### Study design and participants

**Fig. 1.**
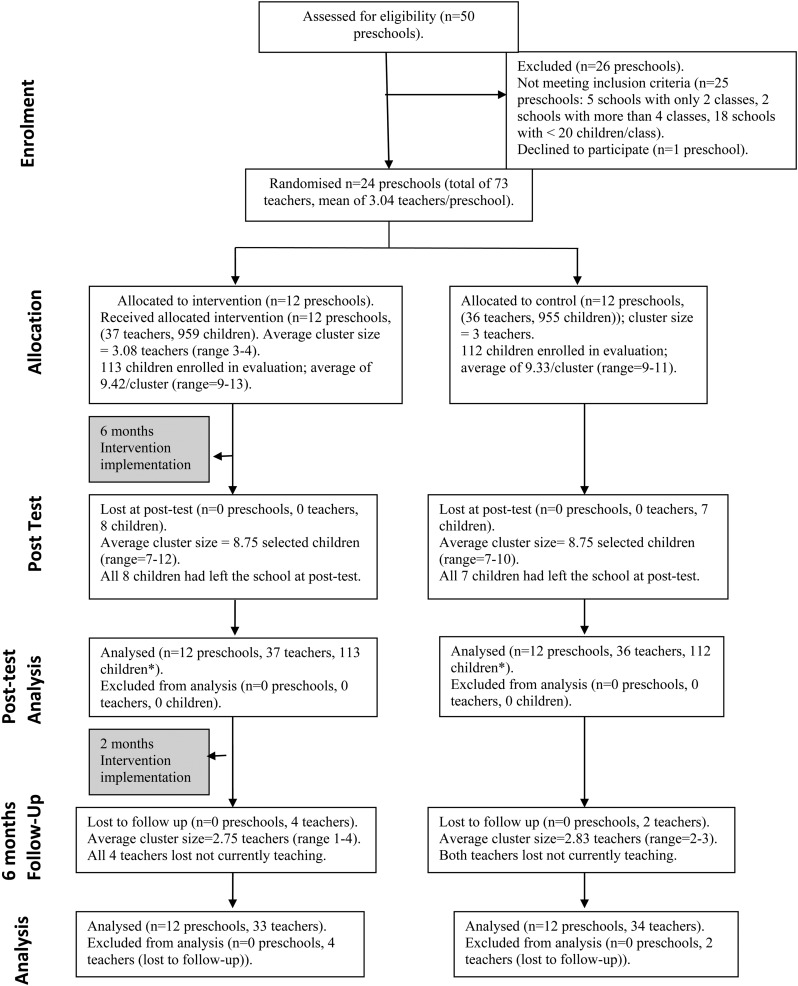
Trial profile. *Analysis was intention to treat; baseline scores substituted for missing post-test data.

The trial took place in 24 community preschools in inner-city areas of Kingston, Jamaica. The preschools were situated in disadvantaged, low SES areas with high levels of community violence. Community preschools cater to children aged 3–6 years and are provided through a partnership between community organisation (often churches) and the government. Parents are expected to provide school resources (e.g. books, pencils, crayons) in addition to paying a small fee. Preschool was the unit of randomisation to prevent contamination between teachers and because the teacher-training intervention was believed to be more effective if all teachers in a school were trained thus leading to changes in the schools’ culture. Preschools were eligible for inclusion if they had 3–4 classes of children, at least 20 children per class, were situated in a specified geographical area and if all teachers consented to participate in the trial. All community schools in three education zones within Kingston were surveyed and in schools with the required number of classes and children, the PI met with the principal to provide information about the study. If the principal was interested in participating in the trial, a further meeting was held with all teachers in the school to describe the study and address any questions and concerns, prior to asking for teachers’ consent. Twenty-four out of fifty preschools approached met all inclusion criteria and were recruited into the study (Fig. 1).

Children with high initial levels of conduct problems were selected for evaluation. An interviewer-administered screening questionnaire was conducted with each class teacher in all study schools. The screen used 10 items from the ICD-10 Diagnostic Criteria for Research for conduct disorder (WHO, 1993), (loses temper, back chats, disobedient/breaks rules, annoys others, blames others, easily annoyed, often angry, spiteful to others, fights or bullies and destroys property), and teachers rated each child in their class according to a four-point scale: not true, just a little true, pretty much true and very much true. The three children with the highest scores on this screen from each class were recruited into the evaluation component of the study. Children were excluded from the evaluation if they had a school attendance <70%, a developmental disability, lived in an institution or if they had a sibling who had already been enrolled into the study. A total of 225 children, aged 3–6 years, were recruited into the study, 113 from intervention schools and 112 from control schools. The focus of this paper is on teacher's behaviour to the whole class and the selected children and to class-wide child behaviour. The effect of intervention on individual child outcomes was reported previously (Baker-Henningham *et al.* 2012).

The University of the West Indies Ethics Committee approved the trial. Written informed consent was obtained from the preschool principals, all teachers and from the parents of the selected children prior to recruitment into the study. The study is registered in the International Standard Randomised Controlled Trial Number Register: ISRCTN35476268.

### Sample size

The study was powered based on the primary outcome of observed child behaviour (Baker-Henningham *et al.* 2012). For measures of teacher's behaviour to the whole class and classroom atmosphere, with 30 teachers per group, a cluster size of three teachers per school and assuming an intra-cluster correlation coefficient of 0.25, we could detect an intervention effect of 1 s.d. with 85% power at 0.05 level of significance. We hypothesised a large ES on these outcomes based on the results of the pilot study (see Table 2). For the measures of teacher behaviour to individual children, with 105 children per group, a cluster size of nine and assuming an intra-cluster correlation coefficient of 0.05, we could detect an intervention effect of 0.5 s.d. with 85% power at 0.05 level of significance.

**Table 2.** Comparison of intervention content and process, teacher attendance and satisfaction, and benefits to teachers and children from adapted and unadapted version of the Incredible Years (IY) teacher-training programme

	Pilot study (five schools)	Current study (24 schools)
Context	11.5% trained teachers	9.6% trained teachers
	23.0% currently attending teacher-training	23.3% currently attending teacher training
	Mean (s.d.) of 23 (5.3) children/class	Mean (s.d.) of 24 (6.6) children/class
	Quality of classroom on ECERS [Mean (s.d.)]:	Quality of classroom on ECERS [Mean (s.d.)]:
	Space and furnishings: 2.62 (0.57)	Space and furnishings: 2.35 (0.69)
	Activities: 2.45 (0.50)	Activities: 1.90 (0.38)
	Interaction: 2.78 (0.60)	Interaction: 2.67 (0.86)
Intervention content	IY teacher-training programme with minor adaptations	IY teacher-training programme with substantial adaptations and additions
	Curriculum unit on social-emotional skills broadly based on IY Dina Dinosaur Classroom Curriculum	Teaching of social and emotional skills was integrated into everyday teaching and learning activities
Intervention Process	Seven full-day teacher-training workshops	Eight full-day teacher-training workshops
	14 lesson unit on social-emotional skills delivered by research team with the teachers’ support (Jan–April)	Four in-class coaching sessions (monthly Jan–Apr)
	Seven in-class consultations (monthly Oct–April)	
Attendance	Median attendance: 6.3 workshops	Median attendance: 8 workshops
	87% attended at least 6 workshops	95% attended at least 6 workshops
	87% received all 21 in-class sessions	89% received all 4 in-class consultations
Satisfaction	Teachers rated all aspects of the training as helpful or very helpful for every workshop	Teachers rated all aspects of the training as helpful or very helpful for every workshop
Benefits at post-test	Effect size [95% confidence interval (CI)]^a^	Effect size (95% CI)^b^
Benefits to teachers		
Teacher positives^c^	3.84 (1.72–5.96)	3.35 (2.70–3.98)
Teacher negatives^c^	−2.02 (−3.84 to −0.08)	−1.29 (−0.87 to −1.71)
Teacher warmth^d^	1.84 (1.16–2.51)	2.03 (1.41–2.67)
Opportunities to share/help^d^	2.22 (0.91–3.53)	5.72 (3.88–7.58)
Benefits to children		
Appropriate behaviour	1.82 (0.31–3.32)	0.73 (0.17–1.30)
Interest and enthusiasm^e^	2.02 (0.24–3.80)	0.98 (0.48–1.50)

a Structured observations and rating scales conducted over four 15-min periods (total of 1 h observation in the morning session only); 27 teachers/classrooms.

b Structured observations and rating scales conducted over 18 five-min periods (total of 90 min observation over whole school day); 73 teachers/classrooms.

c Structured observations of teacher behaviour to the whole class.

d Observer ratings of teacher behaviour to the whole class.

e Observer ratings of class-wide child behaviour.

### Randomisation and masking

Schools were randomised to an intervention or control condition at the start of the intervention year using a computer-generated randomisation sequence by an independent statistician who was blind to the identity of the schools. Children entering the 4- and 5-year-old class in the 2009–2010 academic year were recruited into the study at the end of the previous school year, prior to randomisation. Children entering the 3-year-old class were recruited in October 2009, after randomisation had occurred, by a researcher who was blind to group allocation.

### Intervention

Intervention was implemented at the level of the cluster and involved training all teachers, teacher assistants and principals in the intervention schools using an adapted version of the IY teacher-training programme (Webster-Stratton, 2000). The adaptations for the Jamaican preschool context were based on previous formative and piloting work (Baker-Henningham & Walker, 2009; Baker-Henningham *et al.* 2009a; Baker-Henningham, 2011). The intervention was delivered over one school year through eight full-day training workshops and four in-class support sessions for each teacher. Four of the eight training workshops were conducted on in-service teacher-training days and the intervention teachers attended our workshop rather than attending the standard workshop of the Ministry of Education. Schools also received a small amount of educational materials (e.g. blocks, manipulatives, play doh) to facilitate their use of the strategies taught. At the beginning of the following school year, (after post-test measurements), a refresher intervention was conducted in intervention schools consisting of three additional in-class support sessions. The intervention is described in full in Table 3 using the TIDier checklist (Hoffman *et al*. 2014) and details of the adaptations made are given in Table 1.

**Table 3.** An adapted version of the Incredible Years (IY) teacher-training programme

*Materials and content*
The IY teacher-training curriculum includes content on partnering with parents, developing positive relationships with children, using praise and rewards to motivate children, preventing and managing inappropriate behaviour and teaching social skills, problem solving and anger management in the classroom
During the training, teachers were provided with handouts summarising the key content, cue cards and home-made bingo games to teach and reinforce content related to classroom rules, friendship skills, anger management and emotions, a small hand puppet, stickers and stamps to use as incentives, copies of ‘happygrams’ to share good news with parents and behaviour planning forms. Each school was also provided with a small amount of educational materials (e.g. blocks, manipulatives, play doh) to facilitate their use of the strategies with the children
*Procedures*
The IY teacher-training workshops include the use of videotape modelling, role plays and discussions and follow a collaborative model of training which emphasises participants applying skills and concepts to their own unique situations and engaging in individual and group problem solving. The IY curriculum activities were conducted in the morning sessions of the workshops. During the afternoon sessions, activities focussed on small group work followed by feedback to the larger group. Teachers also received practical classroom assignments to be completed after each workshop. Coaching sessions were aligned with the content covered in the previous workshop and included modelling the strategies, prompting the teacher to use them, providing supportive feedback and helping the teacher to problem-solve any difficulties that arose
*Who provided*
Teacher-training workshops were conducted by the first author who has extensive experience in training teachers and health workers in behavioural interventions. In-class consultations were conducted by a psychology graduate who had experience delivering the intervention during a previous pilot study and who received ongoing supervision and support from the first author
*Where*
Teacher-training workshops were conducted in a church hall in a central location in Kingston. Teachers were given a small stipend to cover transportation costs (US$2/workshop)
*When, how and how much*
Teachers attended eight full-day (6 h) workshops from November to April. A total of 56 intervention school staff were trained in the intervention (including teachers, principals and auxiliary staff). Four of these days were allocated in-service training days and teachers attended in two groups with 26–30 participants per group (i.e. training occurred over eight days). For the remaining four training days, one teacher at a time from each school attended workshop; teachers attended in four groups with 12–16 participants per group (i.e. training occurred over 16 days). In-class assistance and support was also provided to each intervention teacher once a month for four months (Jan–Apr) for approximately 1 h each session
A refresher intervention was provided, (after post-test measurements), in September and October of the following school year consisting of three additional in-class coaching sessions for each teacher. These sessions were designed to reinforce key aspects of the intervention: 1st session was on preventing child behaviour problems by using good classroom organisation skills; 2nd session was on promoting positive child behaviour through positive reinforcement; 3rd session focused on promoting children's social and emotional skills
*Adapting to context*
The teacher-training programme was adapted for the Jamaican preschool context in several ways. We dedicated less workshop time to watching video vignettes followed by discussion and more time on practical activities such as role plays and group work. Role plays and group activities were designed both to teach new concepts and skills and to help teachers practice and generalise previously introduced skills. These activities were based on scenarios common in Jamaican preschools. Card-sorting activities were designed to help teachers learn unfamiliar content (e.g. sorting labelled and unlabelled praise statements). Video vignettes were mostly of Jamaican preschool classrooms, supplemented by the original vignettes as necessary. New simplified handouts and classroom assignments were prepared with content and activities reflecting the Jamaican preschool context. Furthermore, new materials and activities were added to cater to the needs of teachers, many of whom had limited training, with a particular focus on classroom organisation skills and using play and interactive activities in teaching (especially in the context of limited resources, overcrowding and high child/staff ratios). New materials and activities were also designed to help teachers to fully integrate social and emotional skills into daily teaching and learning activities. Detailed notes of teachers strengths and needs were kept during each workshop and during in-class support sessions and these notes were used to inform the content and activities in the ongoing training
*Modifications*
Some modifications were made based on teachers’ individual needs during the in-class coaching sessions. For example, although the coaching was designed to support the content covered in the previous workshop, if a teacher had poor class control, the coach would work flexibly to cover basic classroom management.
*How well* (*planned and actual*)
The workshop facilitator completed a training protocol and self-evaluation after each workshop and the teachers completed workshop evaluations. All of the prescribed content was covered and teachers rated all aspects of the workshops (content, video vignettes, group leader skills and group discussion) as helpful or very helpful for each of the eight workshops. Seventy percent of teachers in the intervention group attended all eight training workshops and 95% attended at least six workshops. 89% of teacher participated in all four in-class consultations

Teachers in control schools attended the standard monthly training workshops provided by the Ministry of Education for all community preschool teachers and received the same educational materials at the same time as the intervention schools.

### Outcome measures

All outcome measures involved independent observations of child and teacher behaviours. Structured observations of teachers’ behaviour to the whole class and to the target children, rating scales of class-wide child behaviour and rating scales of classroom atmosphere were completed at baseline (October–November 2009) and post-test (May–June 2010). A further round of measurements was conducted from May–June 2011 and consisted of all previous measures, except for teachers’ behaviour to the target children. All observational assessments were conducted by researchers blind to the study design, hypothesis and group allocation.

#### Structured observations of teacher behaviour

Observations were conducted over eighteen 5-min periods across one school day and event recording was used to record (1) positive teacher behaviours (use of praise, incentives, describing what children are doing and positive physical behaviours) and (2) negative teacher behaviours (critical comments, negative commands, warnings and negative physical behaviour). The observation schedule and accompanying manual were developed and used previously in Jamaican classrooms (Baker-Henningham *et al.* 2009a). The results for the 18 5-min periods were summed to give the total number of each category of behaviours that occurred during 90 min of observation.

#### Observations of teacher behaviour to the target children

Observations of teacher behaviour to the target children were conducted simultaneously with observations of child behaviour (Baker-Henningham *et al.* 2012). Within each class, three children were observed for 5 min each on a rotational basis for a total of 15 min each day per child over 4 days, across different times of the school day giving a total of 1 h of observation per child. Event recording was used for positive behaviours to the target child (e.g. positive attention, praise, incentive) and negative behaviours (e.g. critical comment, negative command) and the results were summed to give the total in 1 h of observation. All behaviours were defined in a manual.

#### Rating scales of child behaviour and the classroom atmosphere

Two ratings of the behaviour of the children in the class as a whole: level of appropriate behaviour (e.g. no fighting or other aggressive behaviours, children are not disrupting other students), and level of interest and enthusiasm displayed by children (e.g. children are enjoying classroom activities) and two ratings of teacher's behaviour: teacher provides opportunities for children to share and help each other and teacher warmth were developed based on scales used by the Conduct Problems Prevention Research Group (1999) and used previously in Jamaica (Baker-Henningham *et al.* 2009a). Ratings were completed at the end of each 5-min teacher observation period using five-point scales (1–5, where 1 is low and 5 is high). The average rating over the 18 observation periods was calculated.

### Other measures

#### Quality of the classroom environment

The quality of the classroom environment was assessed at baseline only using three subscales from the Early Childhood Education Rating Scales – Revised (ECERS-R) (Harms *et al.* 1998). The ECERS is a commonly used comprehensive measure of early childhood classrooms and higher scores on the ECERS have been shown to be associated with improved child development in several studies (Mashburn *et al.* 2008). The following subscales were used:

*Space and furnishings*: the adequacy and arrangement of the classroom area and the appropriateness of the furniture (eight items);

*Language and reasoning*: provision of books and pictures and a range of communicative activities (four items);

*Activities*: children's involvement in a range of developmentally appropriate activities (e.g. sand and water, block play, dramatic play, fine motor skills) and the variety of opportunities provided within each activity (eight items).

Scores on each subscale range from 1 to 7 with 1 indicating inadequate quality, 3 indicating minimal quality, 5 indicating good quality and 7 indicating excellent quality. In each classroom, the ECERS-R was completed during a 3 h observation period followed by a 20- min interview with the teacher. The observations were conducted by a single observer. Inter-observer reliability between the observer and the trainer were >0.90 for each subscale on 10 consecutive measures prior to the start of the study.

### Quality of measures and masking

For all observational data, intra-class correlation coefficients (ICCs) between the observers and trainer and between each pair of observers were calculated for 20 consecutive 5-min observations prior to data collection at each time point and for 10–15% of observations during ongoing data collection. The ICCs during training were: median (range) = 0.89 (0.81–0.97) at baseline, 0.92 (0.86–0.99) at post-test and 0.89 (0.85–0.96) at follow-up. Ongoing reliabilities were: median (range) = 0.90 (0.86–0.93). (Table 4).

**Table 4.** Inter-observer reliabilities for each outcome measurement over 5 min intervals [median (range)]

	Inter-observer reliabilities (using ICCs)			
	Baseline^a^	Post-test^a^	Follow-up^a^	Ongoing^b^
Structured observations of teacher behaviour to the whole class				
Teacher positives	0.97 (0.94–0.99)	0.99 (0.99–1.00)	0.96 (0.90–0.98)	0.91 (0.79–0.95)
Teacher negatives	0.94 (0.90–0.97)	0.93 (0.83–0.97)	0.92 (0.84–0.95)	0.93 (0.80–0.96)
Structured observations of teacher behaviour to target children				
Teacher positives	0.90 (0.86–0.94)	0.93 (0.86–0.97)	–	0.92 (0.83–0.98)
Teacher negatives	0.86 (0.78–1.0)	0.86 (0.72–1.0)	–	0.91 (0.86–0.96)
Rating Scales of teacher behaviour to the whole class				
Teacher warmth	0.84 (0.65–0.93)	0.93 (0.86–0.97)	0.88 (0.82–0.92)	0.87 (0.72–0.94)
Opportunities to share/help	0.96 (0.92–0.99)	0.93 (0.82–0.97)	0.86 (0.80–0.98)	0.90 (0.82–0.95)
Rating Scales of class-wide child behaviour				
Appropriate behaviour	0.87 (0.73–0.95)	0.86 (0.82–0.92)	0.88 (0.79–0.95)	0.86 (0.82–0.92)
Interest and enthusiasm	0.81 (0.60–0.92)	0.90 (0.79–0.96)	0.85 (0.71–0.92)	0.86 (0.80–0.92)

ICC, intra-class correlation coefficient.

a *n* = 20 observations prior to starting data collection between trainer and each observer and a minimum of 15 between each observer and other observers.

b 15% of all observations between master coder and each observer and a minimum of 10 between each observer and other observers during each data collection period.

Five researchers conducted the observations of the teachers’ behaviour to the target children and three researchers conducted observations of teacher behaviour to the whole class, classroom atmosphere and class-wide child behaviour. Researchers conducted equal numbers of measurements from each group and were blind to the study design and group allocation. To maintain blindness of researchers, teachers were asked at initial contact and prior to each assessment not to reveal intervention status and observers were not informed of the study design, hypothesis or group allocation and were employed during the measurement phases of the study only.

### Statistical analysis

The distributions of continuous dependent variables were examined for normality. Normality was rejected for observations of teacher's positive and negative behaviours to the whole class and to target children and for ratings of class-wide child behaviour at baseline and post-test. The two variables measuring teacher's behaviour to the whole class were normalised using a square root transformation, a log transformation was used for the variables measuring teachers’ behaviour to the target children, while the class-wide child behaviour rating was normalised by squaring.

The effect of intervention was examined using multilevel multiple regression models to take into account the hierarchical nature of the data (Hayes & Moulton, 2009). For the analyses of the effect of intervention on teacher's behaviour to the whole class, class-wide child behaviour outcomes and ratings of classroom atmosphere at post-test, baseline score and intervention group were entered as fixed effects and school was added as a random effect. In the analyses on teacher behaviour to target children, child age and sex, baseline score and group were entered as fixed effects and school and teacher were entered as random effects. All outcomes at follow-up were at the level of the classroom/teacher; group was entered as a fixed effect and school as a random effect. All multilevel analyses were conducted with MLwiN version 2.10 (Rasbash *et al.* 2009).

## Results

### Sample

All 24 preschools were followed up at post-test and 6-month follow-up and all 73 teachers were followed up at post-test. Six teachers (8.2%) were lost at 6-month follow-up (four intervention, two control) and there were no significant differences on any outcome measures at baseline or post-test between those lost *v.* those retained. However, teachers lost were significantly younger (*p* = 0.02) and had been teaching for fewer years (*p* = 0.006). There were no significant differences between the intervention groups at baseline although number of years teaching in current school was marginally significant (*p* = 0.06). (Table 5).

**Table 5.** Child, classroom, teacher and school characteristics by study group: Values are mean (s.d.) unless otherwise stated

	Intervention	Control	*p* value
Child characteristics	*n* = *113*	*n* = *112*	
Child age (in years)	4·2 (0·9)	4·2 (0·8)	0·86
Child sex: *n* (%) boys	67 (59·3%)	71 (63·4%)	0·53
Teacher characteristics	*n* = *37*	*n* = *36*	
Age of teacher (in years)	38·2 (10·7)	42·8 (9·8)	0·20
Number of years teaching	12·6 (9·0)	13·8 (8·1)	0·65
Number of years teaching at current school	7·9 (7·1)	11·4 (6·1)	0·06
Number of children in class	23·2 (6·9)	25·2 (6·4)	0·19
Teacher completed high school *n* (%)	26 (72.2%)	30 (81.1%)	0·37
Trained teacher: *n* (%)	4 (10·8%)	3 (8·3%)	0·72
Currently attending teacher-training college: *n* (%)	8 (21·6%)	9 (25·0%)	0·73
Sex of teacher: *n* (%) female	34 (91·9%)	35 (97·2%)	0·32
Classroom characteristics	*n* = *37*	*n* = *36*	
Number of children in class	23·2 (6·9)	25·2 (6·4)	0·19
ECERS-R^a^: Space and furnishings subscale	2.33 (0.69)	2.38 (0.70)	0·76
ECERS-R^a^: Activities subscale	1.90 (0.37)	1.99 (0.39)	0·29
ECERS-R^a^: Interaction subscale	2.59 (0.91)	2.81 (0.98)	0·34
School characteristics	*n* = *12*	*n* = *12*	
Average school attendance in 1st term [mean % (s.d.)]	80·5 (6·4)	77·9 (7·9)	0·39
Number of children enrolled	71·2 (22·5)	75·7 (10·8)	0·54

a Early childhood environment research scale-revised.

Few teachers were qualified teachers (9.5%) although almost a quarter were currently attending teacher-training college. The teachers had been teaching for an average of 13 years and were predominantly female (94.5%). The quality of the classrooms as measured by the ECERS was generally in the inadequate range (<3) indicating that the physical conditions of the classrooms were poor (e.g. overcrowded, inappropriate furniture) and play and learning materials were extremely limited.

### Findings

Raw scores for all outcomes by group at baseline, post-test and 6-month follow-up are given in Table 6. There were no significant differences between the groups at baseline.

**Table 6.** Raw scores of structured observations of teachers’ behaviour, ratings of class-wide child behaviour and ratings of teacher behaviour at baseline and post-intervention by intervention group

	Intervention (*n* = 37)			Control (*n* = 36)		
	Baseline (*n* = 37)	Post-test (*n* = 37)	Follow-up (*n* = 33)	Baseline (*n* = 36)	Post-intervention (*n* = 36)	Follow-up (*n* = 33)
Structured observations of teacher behaviour to the whole class^a^						
Teacher positives [median (range)]	52 (0–256)	228 (18–726)	145 (5–528)	63 (18–141)	45 (6–157)	37 (10–163)
Teacher negatives [median (range)]	98 (21–231)	58 (22–165)	68 (6–191)	99 (39–231)	105 (34–201)	113 (14–262)
Structured observations of teacher behaviour to target children^b^						
Teacher positives [median (range)]	9 (1–37)	22 (2–92)	–	11 (0–47)	10 (0–63)	–
Teacher negatives [median (range)]	8 (0–27)	5 (0–30)	–	9 (0–47)	9 (0–54)	–
Rating scales of class-wide child behaviour^c^						
Appropriate behaviour [median (range)]	3.60 (1.78–4.50)	4.22 (2.11–4.89)	3.84 (0.50)^d^	3.60 (2.39–4.83)	3.94 (2.28–4.67)	3.51 (0.11)^d^
Interest and enthusiasm [mean (s.d.)]	2.82 (0.53)	3.90 (0.44)	3.42 (0.54)	3.03 (0.46)	3.42 (0.41)	3.13 (0.36)
Rating scales of classroom atmosphere^c^						
Teacher warmth [mean [s.d.)]	2.44 (0.46)	3.66 (0.62)	2.93 (0.47)	2.69 (0.53)	2.77 (0.61)	2.53 (0.44)
Opportunities provided to share and help [mean (s.d.)]	2.02 (0.09)	2.87 (0.53)	2.21 (0.31)	2.07 (0.14)	2.22 (0.26)	2.04 (0.12)

a Event sampling over 18 5-min observation periods to give a total of 90 min of observation.

b *n* = 113 intervention; *n* = 112 control; Event sampling over 12 5-min periods over four school days to give a total of 1 h of observations.

c Observer ratings over 18 5-min observations periods over 1 school day on a scale of 1–5, where 1 is low and 5 is high.

d Mean (s.d.) (median (range) was used for non-normally distributed variables).

At post-test (Table 7), significant benefits of intervention were found for the number of positive and negative teacher interactions to the whole class (positive: ES = 3.35; negative: ES = −1.29) and the number of positive and negative teacher interactions to the high-risk children (positive: ES = 0.83; negative: ES = −0.50). Furthermore, teachers in intervention classrooms were rated as being more warm (ES = 2.03) and providing more opportunities for children to work together (ES = 5.72). Significant benefits of intervention were also found for class-wide measures of child behaviour including the level of appropriate child behaviour (ES = 0.73) and children's interest and enthusiasm (ES = 0.98).

**Table 7.** Multilevel regression analyses of effect of intervention on teacher behaviour to the whole class and classroom ratings and teacher behaviour to the target child

	*B* (95% CI)	ICC^a^	Effect size (95% CI)	*p* value
Structured observations of teacher behaviour to the whole class				
Teacher positive behaviours^b^^,^^c^	8.26 (6.67–9.85)	0.13	3.35 (2.70–3.98)	<0.0001
Teacher negative behaviours^b^^,^^c^	−2.57 (−3.41 to −1.73)	0.03	−1.29 (−0.87 to −1.71)	<0.0001
Structured observations of teacher behaviours to target child				
Positive behaviours^d^^,^^e^	0.25 (0.15–0.35)	0.07	0.83 (0.50–1.17)	<0.0001
Negative behaviours^d^^,^^e^	−0.15 (−0.23 to −0.07)	0.015	0.50 (0.23–0.77)	0.0004
Ratings of class-wide child behaviour				
Level of children's appropriate classroom behaviour^b^^,^^f^	3.34 (0.76–5.91)	0.49	0.73 (0.17–1.30)	0.01
Level of children's interest nd enthusiasm^b^	0.49 (0.24–0.75)	0.30	0.98 (0.48–1.50)	0.001
Rating scales of classroom atmosphere				
Opportunities to share & help each other^b^	0.69 (0.47–0.91)	0.18	5.72 (3.88–7.58)	<0.0001
Teacher warmth^b^	1.04 (0.72–1.36)	0.38	2.03 (1.41–2.67)	<0.0001

a Intra-cluster correlation coefficient.

b Analysis adjusting for baseline score as a fixed effect and school as a random effect.

c Analysis adjusting for baseline score, child age and sex as fixed effects and school and classroom as random effects.

d Square root of raw score used in the analysis.

e Log of raw score used in the analysis.

f Square of raw score used in the analysis.

At 6-month follow-up (Table 8), significant benefits to all measures were sustained. Intervention teachers continued to have significantly more positive interactions (ES = 2.70) and fewer negative interactions (ES = −0.98) with the whole class and were rated significantly higher on warmth (ES = 0.91) and on provision of opportunities for children to share and help each other (ES = 1.42). The ratings of class-wide child behaviour showed significant benefits to the level of appropriate behaviour (ES = 0.5) and the level of interest and enthusiasm (ES = 0.78).

**Table 8.** Multilevel regression analyses of effect of intervention on teacher behaviour to the whole class and classroom ratings at 6-month follow-up

	*B* (95% CI)	ICC^a^	Effect size (95% CI)	*p* value
Structured observations of teacher behaviour to the whole class				
Teacher positive behaviours^b^	6.33 (4.68–7.98)	0.05	2.70 (2.0–3.41)	<0.0001
Teacher negative behaviours^b^	−2.46 (−3.61 to −1.31)	0.02	−0.98 (−0.52 to −1.44)	<0.0001
Ratings of class-wide child behaviour				
Level of children's appropriate classroom behaviour	0.32 (0.02–0.62)	0.13	0.50 (0.03–0.97)	0.04
Level of children's interest and enthusiasm	0.28 (0.01–0.55)	0.26	0.78 (0.03–1.53)	0.04
Rating scales of classroom atmosphere				
Opportunities to share and help each other	0.17 (0.06–0.28)	0.02	1.42 (0.5–2.33)	0.003
Teacher warmth	0.40 (0.15–1.00)	0.00	0.91 (0.46–1.43)	0.002

All analyses adjust for school as a random effect.

a Intra-cluster correlation coefficient.

b Square root of raw score used in the analysis.

### Comparison to pilot study

Table 2 compares the context, intensity, satisfaction and benefits of the current study with a pilot study we conducted previously. In the pilot study, the IY teacher-training intervention was implemented with few adaptations, unlike in the current study in which significant changes were made. The proportion of trained teachers and class sizes were similar in both studies. The schools in the pilot study scored 0.43 s.d. higher on the space and furnishings subscale of the ECERS and 1.25 s.d. higher on the activities subscale indicating a greater level of resources. However, ECERS scores for both studies were in the minimal range. The intervention used in the pilot study was more intensive and included seven full-day workshops and a total of 21 in-class support sessions with each teacher with 14 of these also working directly with the children teaching a curriculum unit on social and emotional skills (in collaboration with the teacher); in the current study the number of workshops was similar (8 full-day workshops) but teachers received only four in-class support sessions. In both the pilot study and the current study, the intervention was acceptable to teachers as shown by attendance and satisfaction ratings and large benefits were found to teacher behaviour and class-wide child behaviour.

## Discussion

An adapted version of an evidence-based programme aimed at training teachers in appropriate classroom management techniques in order to reduce child conduct problems and promote child competencies led to large benefits to Jamaican preschool teachers’ behaviour to the whole class and to children at high risk for developing conduct problems, to class-wide child behaviour and to classroom atmosphere. These benefits were sustained at 6-month follow-up.

The study shows that professional development training in classroom behaviour management for preschool teachers in LMIC has the potential to make large improvements to teachers’ behaviours resulting in a more emotionally supportive classroom environment and reduced harsh punishment by teachers for all children and improved class-wide child behaviour. Violence against children in schools is common in many LMIC and yet the evidence base for effective interventions is sparse. We are aware of only one trial: in primary schools in Uganda, a school-wide intervention involving staff, students and the school administration reduced past week physical violence against children by 42% (Devries *et al.* 2015). Importantly, benefits in the current study were found not only for teachers’ behaviour to the whole class but also to teachers’ behaviour to children at high risk for developing conduct problems. Increased positive attention and reduced negative attention to these children may have limited the escalating cycles of coercive behaviour between teachers and antisocial children (Patterson *et al.* 1992), and this is likely to be a key mechanism in the reductions in child conduct problems and increases in social skills found for these children (Baker-Henningham *et al.* 2012). In addition, due to the benefits to class-wide child behaviour, children at high risk for developing conduct disorder in intervention schools would be exposed to less aggression and increased appropriate behaviour from peers which may have also contributed to their improved behaviour at school and at home (Kellam *et al.* 1998). Benefits were also found to class-wide child interest and enthusiasm in learning activities and this increased participation is likely to lead to greater school bonding and increased learning, which are key predictors of improved behaviour (O'Donell *et al.* 1995). Benefits to teachers’ behaviours and to class-wide child behaviour were sustained at a 6-month follow-up. This trial is important to the global agenda on violence prevention in two main ways: (i) by reducing harsh punishment by teachers concurrently and at 6-month follow-up and (ii) by reducing conduct problems in children at high-risk of developing antisocial behaviour thus reducing future violence through early prevention. The sustained benefits of the intervention are promising as if teachers continue using the strategies, there are potential benefits to new cohorts of children. It will be important to determine if the benefits to teacher practices from interventions such as this are sustained over the long term and how frequently booster-training sessions may be required to ensure teachers continue to use the skills.

Benefits to teacher behaviour to the whole class were large compared to previous studies. For example, benefits from the IY teacher-training programme in the USA have been in the range 0.49–0.67 for ratings of harsh/critical practices and 0.51 for positive practices (Webster-Stratton *et al.* 2001, 2008), although large benefits were found for ratings of social-emotional teaching (ES = 0.96) and effective discipline (ES = 1.24) (Webster-Stratton *et al.* 2008). In the meta-analysis by Werner *et al.* (2016), training caregivers in early childhood settings led to medium effect sizes for caregiver–child interaction (ES = 0.44) and classroom quality (ES = 0.39). Benefits were likely larger in the current study due to the low level of initial training of the teachers thus widening the differences between the groups with intervention. For example, the largest effect sizes were found for the teacher behaviours that were less common in the Jamaican preschool context (teacher positives to the whole class and teacher provides opportunities to share and help). This explanation is also supported by the finding that in the IY teacher-training programme in the USA, effects sizes on critical behaviour for teachers more than 2 s.d. above the mean at baseline were 1.37 (compared to 0.49 for the entire sample) (Webster-Stratton *et al.* 2008). One study examined benefits of teacher training and consultation on teachers’ behaviour to children at high risk for aggression and found benefits to the amount of teacher feedback to these high-risk children with effect sizes of 0.46–0.62 (The Metropolitan Area Child Study Research Group & Gorman-Smith, 2003), which are in a similar range to those found in this study.

This study also adds to the debate about the transportability of evidence-based child mental health interventions. Although we are aware of no studies empirically investigating the transportability of teacher interventions across cultures and contexts, meta-analytical studies of parenting interventions have mixed results with van Mourik *et al.* (2017) reporting increased effectiveness for ethnic minority families if interventions are adapted to fit the cultural context, whereas Gardner *et al.* (2016) found that interventions were at least as effective when transported across countries, suggesting that extensive adaption is not required. We chose to use the IY teacher-training programme as its collaborative nature makes it inherently adaptable and culturally sensitive and the IY programmes have been shown to be effective in multiple countries and with a range of ethnic groups (Menting *et al.* 2013). However, despite this in-built cultural flexibility, we found that an adapted version of the programme required significantly less in-class support for teachers than a minimally adapted version (4 sessions *v.* 21 sessions), to produce benefits of a similar magnitude to teachers’ classroom practices. Teacher attendance and satisfaction was high with both the minimally adapted and significantly adapted version indicating that participant engagement was not the determining factor. The adaptations were aimed at promoting teacher understanding, confidence and ability to use the strategies in various situations across the school day and ensuring the skills and strategies were introduced in an ecologically valid way (i.e. in a way that was feasible in the Jamaican preschool context). This increased the likelihood that teachers could successfully implement the strategies in the classroom without extensive in-class support and is important for implementation costs if the programme is to be widely disseminated. Other factors that need to be considered in transporting evidence-based interventions are the costs associated with the programme (both the upfront cost involved in purchasing the materials and any ongoing training costs) and its acceptability to policymakers and these factors are key for local adoption of the programme by the Ministry of Education and for ongoing sustainability.

It should be noted that caution needs to be exercised in comparing the results from this study to the results from the pilot study. The trials differed in several ways including different sampling criteria (the pilot study included two larger better resourced schools and three more typical schools), different lengths of observations (observations were conducted in the morning session only in the pilot study and for the whole day in the current study) and in the pilot study the teacher-training programme was supplemented with a structured curriculum unit on social–emotional skills.

The study has several strengths. The trial had high response rates and attrition was low. The randomisation led to balanced groups at baseline with no significant differences between the groups on any variable. All measurements involved independent observations which are less open to bias than teacher reports of their own behaviour, and observations were conducted by research assistants who were blind to the study design, hypothesis and group allocation. Furthermore, the trial included a 6-month follow-up showing that benefits were sustained after the end of the teacher-training period. There are also several limitations. Teachers were aware of their intervention status and it is possible that they behaved differently during observation. However, there is evidence that conducting observations over the whole day increases the reliability of data and minimises reactivity to being observed (Stoolmiller *et al.* 2000). In addition, community preschools in Jamaica have poor physical conditions including overcrowding, high noise levels, high child–teacher ratios and usually have several classrooms in a shared space and in this context, the observer is unobtrusive. Contamination between groups was minimised through the use of a cluster design; however, it is possible that some sharing occurred as schools were situated in a common geographical area, and over 20% of teachers in both groups were enrolled in teacher-training colleges; however, this would reduce the differences between the groups. The generalisability of the results may be limited by the fact that we excluded schools with fewer than 20 children per class and schools with fewer than three and more than four classrooms. However, as the intervention is delivered to all teachers within the school, we anticipate that teachers in smaller and larger schools would also benefit.

## Conclusions

We found large, significant benefits to teachers’ behaviour and class-wide child behaviour from an intervention providing teachers with training in classroom behaviour management and in how to promote children's social–emotional competence. These benefits were assessed by independent, masked observers and sustained at 6-month follow-up. The intervention also led to significant benefits to the behaviour of children at high risk for developing conduct problems at home and at school (see Baker-Henningham *et al.* 2012). Furthermore, we report that making substantial adaptations to an evidence-based programme to ensure contextual fit reduced the amount of individual support teachers need in order to use the skills effectively. We used the IY teacher-training programme and the content was relevant and appropriate to the Jamaican context and we believe that the majority of the strategies introduced through the programme are applicable across different cultures and contexts. However, we also found that adaptations to the materials and methods used improved the cultural and contextual fit and we tentatively conclude that although the strategies are generally relevant and applicable, how they are operationalised in practice is likely to differ across different cultures, contexts and situations. The intervention was implemented through the existing educational services and involved training existing staff and hence should be feasible and sustainable at scale. Training teachers has the advantage that multiple high-risk children can be helped and teachers can continue benefiting new cohorts of children over time. The findings have important implications for child mental health in low-resource settings and show that teacher-training programmes are an important component of initiatives to prevent violence against children, promote child well-being and prevent child mental health problems in LMIC.
